# Autophagy Contributes to Host Immunity and Protection against Zika Virus Infection via Type I IFN Signaling

**DOI:** 10.1155/2020/9527147

**Published:** 2020-04-28

**Authors:** Yuyi Huang, Yujie Wang, Shuhui Meng, Zhuohang Chen, Haifan Kong, Ting Pan, Gen Lu, Xuefeng Li

**Affiliations:** ^1^The Sixth Affiliated Hospital of Guangzhou Medical University, Qingyuan People's Hospital; The Second Affiliated Hospital of Guangzhou Medical University, The State Key Laboratory of Respiratory Disease, Guangdong Provincial Key Laboratory of Allergy & Clinical Immunology; Sino-French Hoffmann Institute, School of Basic Medical Sciences, Guangzhou Medical University, Guangzhou 511436, China; ^2^School of Public Health (Shenzhen), Sun Yat-sen University, Shenzhen 510006, China; ^3^Nan Shan School, Guangzhou Medical University, Guangzhou 511436, China; ^4^School of Medicine, Sun Yat-sen University, Guangzhou 510080, China; ^5^Guangzhou Women and Children's Medical Center, Guangzhou Medical University, Guangzhou 510120, China; ^6^Shenzhen Luohu People's Hospital, The Third Affiliated Hospital of Shenzhen University, Shenzhen 518001, China; ^7^Key Laboratory of Regenerative Biology, Guangdong Provincial Key Laboratory of Stem Cell and Regenerative Medicine, South China Institute for Stem Cell Biology and Regenerative Medicine, Guangzhou Institutes of Biomedicine and Health, Chinese Academy of Sciences, Guangzhou 510530, China

## Abstract

Recent studies have indicated that the Zika virus (ZIKV) has a significant impact on the fetal brain, and autophagy is contributing to host immune response and defense against virus infection. Here, we demonstrate that ZIKV infection triggered increased LC3 punctuation in mouse monocyte-macrophage cell line (RAW264.7), mouse microglial cell line (BV2), and hindbrain tissues, proving the occurrence of autophagy both *in vitro* and *in vivo*. Interestingly, manual intervention of autophagy, like deficiency inhibited by 3-MA, can reduce viral clearance in RAW264.7 cells upon ZIKV infection. Besides, specific siRNA strategy confirmed that autophagy can be activated through Atg7-Atg5 and type I IFN signaling pathway upon ZIKV infection, while knocking down of Atg7 and Atg5 effectively decreased the ZIKV clearance in phagocytes. Furthermore, we analyzed that type I IFN signaling could contribute to autophagic clearance of invaded ZIKV in phagocytes. Taken together, our findings demonstrate that ZIKV-induced autophagy is favorable to activate host immunity, particularly through type I IFN signaling, which participates in host protection and defense against ZIKV infection.

## 1. Introduction

Zika virus (ZIKV) is a flavivirus that was first detected in Uganda and named after the Zika forest. It was first discovered in the rhesus monkey in 1947 and in humans in 1952 [1, 2]. The virus spread is mainly dependent on mosquitoes and has been confirmed to occur in 49 countries or regions worldwide [3]. People at the early stage after ZIKV infection usually presented with mild fever like in dengue infection, while a series of conditions and adverse symptoms in the later period [4]. A case analysis revealed that ZIKV infection may cause Guillain-Barre syndrome (GBS) [5], congenital Zika syndrome (CZS) and uveitis [6, 7], myelitis and meninges encephalitis [8, 9], microcephaly [10], and other brain-related diseases. ZIKV has been detected in the urine, serum, amniotic fluid of pregnant women, and brain of fetuses with microcephaly [11, 12]. It is confirmed that after the outbreak of the ZIKV epidemic, multiple cases of febrile rash in pregnant women associated with ZIKV were reported, which indicated that the virus can cross the placental barrier and infect the newborn brain tissue [13].

Autophagy is an ancient self-eating phenomenon in mammalian cells that has numerous effects on innate immunity. It affects inflammation through regulatory interactions with immune signaling [14]. Autophagy also mediates multiple aspects of the immune response involving direct digestion and degradation of intracellular pathogens [15], thus plays an important role in the battle with viruses and mediating their elimination [16]. Besides innate immunity, autophagy coordinates with adaptive immunity in the host antiviral response [17]. Thus, the immune system uses autophagy as both a monitor for evidence of pathogen invasion and cellular transformation and an effector mechanism to clear intracellular pathogens [18]. However, the interference of virus formation with autophagosome or fusion with lysosome is a double-edged sword during viral infection [19–22]. A previous study indicates that ZIKV infection can induce autophagy in human umbilical vein cells (HUVEC) and human trophoblastic cells, and inhibition of ZIKV-induced autophagy restrains viral replication [23, 24]. ZIKV infection also induces mitosis abnormalities and apoptotic cell death in human neural progenitor cells [25]. Previous reports have shown inflammatory autophagy as an antiviral innate defense mechanism against ZIKV infection, which plays a role in the control of viral infection [26].

To explore whether autophagy is a possible therapeutic target to counteract ZIKV infection [27], we used ZIKV-infected mice and cell models to study whether autophagy plays roles in viral phagocytosis, clearance, and host immunity of antiviral immune response. We found that autophagy protects the mice or cell from severe infection through type I IFN signaling, and manipulation of autophagy both in vitro and in vivo could help the host immunity against ZIKV infection. Altogether, our findings showed new insights into the mechanism related to activate host immune response and autophagy participates in host protection and defense against ZIKV infection.

## 2. Materials and Methods

### 2.1. Mouse

SJL mice were bought from Charles River in Beijing, China. C57BL/6J mice were bought from Chase Ray in Guangzhou, China. Mice were kept and bred in the animal facility of Guangzhou Medical University. Mice were infected with ZIKV (1 × 10^6^ PFU/mouse) by tail vein injection on the seventh day of pregnancy, and newborn mice were sacrificed after birth for brain tissue histological research and other procedures. The hindbrain, midbrain, and forebrain were excised for homogenization or fixed in 10% formalin. The study is approved by Guangzhou Medical University experimental animal ethics committee.

### 2.2. Cell Culture

RAW264.7 cells, BV2 cells, and Vero cells were bought from ATCC. The genotypes of all the cell lines were verified, and the cells were mycoplasma free. RAW264.7 cells and BV2 cells were maintained in RPMI 1640 medium with 10% newborn calf serum and 100 U/ml penicillin-streptomycin (P/S) antibiotics in a 5% CO_2_ incubator. Vero cells were cultured in a medium (DMEM) supplemented with P/S and 10% newborn calf serum at 37°C with 5% CO_2_. Rapamycin and 3-MA were bought from Selleck. Before ZIKV infection, cells were treated with rapamycin for 12 hours and 3-MA for 3 hours, respectively. SiRNA knockdown and CRISPR knockout were performed following the manufacturer's instructions (ATG7 siRNA, sc-41448, ATG5 siRNA, sc-41446, IFNAR1 siRNA, sc-40090, IFNAR2 sc-40092, sc-40092, IFNGR1 CRISPR Plasmids, sc-401191, IFNGR2 siRNA, sc-35635, IL10R2 siRNA, sc-75332, IFNLR1 siRNA, and sc-62498).

### 2.3. ZIKV Strains

ZIKV strain MR766 (rhesus/1947/Uganda) and PRVABC59 were generously provided by Dr. Bishi Fu from Harvard University. We isolated the ZIKV SYSU/2016 strain from the urine of a man who had traveled from Venezuela to China in March 2016 and amplified it in C6/36 cells or the brains of suckling mice after intracerebroventricular injection. Phylogenetic analysis of the whole genome indicated that this ZIKV strain is closely related to Brazilian and other South American isolates and belongs to the Asian lineage rather than to the African lineage [28]. ZIKV stocks were propagated in Vero cells inoculated at an MOI of 0.02, and supernatants were harvested at 96 h postinfection.

### 2.4. PFU Assay

In virus titer determination and plaque assay, Vero cells were inoculated on a 6-well plate at a density of 10^6^/well on the day before the experiment. In wild-type ZIKV titration and plaque assay, the titration of wild-type ZIKV was performed by a plaque assay by following the procedures described previously with minor modifications (48). Briefly, Vero cells were seeded in 12-well plates and used for infection when the cells were grown to 100% confluence. For phagocytosis assay, after 1 h infection, the macrophage was washed with phosphate-buffered saline (PBS) once and lysed; as for phagocytosis assay, 1 h after infection is considered a short-term treatment to macrophage, in which the phagocytosis is going as a major process. After 1 h infection, the floating viruses in the cell surface and outside were removed, the cells were washed with PBS, and the fresh medium was changed. After another 11 h, the cells do not phagocyte and only digest the internalized virus. Thus, 12 h after infection, the survived virus is considered the defeat of clearance, and the PFU assay is suitable for clearance ability in our model. Then, the macrophage was collected and moderately physically lysed using a grinding rod. For tissue viral burden, the fresh brain tissues were collected and grinded moderately. The lysates were diluted by PBS with 10^−1^, 10^−2^, 10^−3^, 10^−4^, and 10^−5^, respectively. Vero cells were infected with the above lysates including living viruses for 1 h in 37°C incubator supplied with 5% CO_2_. The cells were overlaid with agarose-DMEM containing 0.6% bovine serum albumin (BSA) and 1% low-melting-point agarose. The contents of the plates were settled to 4°C for 10 min until the agarose medium became solid, then cultured upside down at 37°C incubator for 5-7 days. The virus titers were determined by counting visible plaques. Data are shown as means ± SD from three independent experiments [28].

### 2.5. Cell Transfection

Cells were transfected with Atg5 small-interfering RNA (siRNA; Invitrogen) or Atg7 siRNA using Lipofectamine 2000 reagents (Invitrogen) in serum-free RPMI 1640 medium following the manufacturer's instructions.

The tandem GFP-LC3 plasmid was made and obtained from Tamotsu Yoshimori of Osaka University, Japan. Cells were grown in glass-bottomed dishes and transfected with tandem GFP-LC3 plasmids for 24 h using Lipofectamine 2000 reagent (Invitrogen) in serum-free RPMI 1640 medium (Thermo Fisher Scientific) following the manufacturer's instructions. For fluoresce observation, after different infections, the cells were fixed in 4% paraformaldehyde. 100 randomly selected cells in each group were counted for visible LC3 GFP dots to compare autophagy occurrence. Data are shown as the representative of captured images [15, 29].

### 2.6. Western Blotting

Rabbit polyclonal antibody against MAP LC3b, goat polyclonal antibody against beclin-1, and rabbit monoclonal antibody against GAPDH were bought from Proteintech. The samples from cells or tissues were lysed and quantified. The lysates were boiled for 5 minutes then separated by 10% SDS-polyacrylamide gel electrophoresis (PAGE). The proteins were then transferred to nitrocellulose membranes after electrophoresis and then blocked membranes for 2 h with 5% skimmed milk blocking buffer. Membranes were incubated with first antibodies following the instructions of manufacturer. After washing three times with a washing solution, the membranes were incubated with a secondary antibody. Signals were visualized using an enhanced chemiluminescence detection kit.

### 2.7. Confocal Microscopy and Indirect Immunofluorescence Staining

For fluoresce observation, after infection, the tissues were fixed in 4% paraformaldehyde and then sectioned for histological analysis. The tissues were permeabilized with 0.2% Triton X-100 in PBS. After blocking with a blocking buffer for 30 minutes, tissues were incubated with primary antibodies at 1 : 500 dilution in the blocking buffer and incubated overnight. The primary antibody to Zika Virus Envelope (E) Protein (D1-4G2-4-15) antibody was bought from Kerafast Boston, MA. After incubation for 1.5 h in 37°C incubator, the tissue sections were washed three times with washing buffer and incubated with appropriate fluorophore-conjugated secondary antibodies. Finally, the sections were washed and prepared for immunofluorescence observation as previously described [30]. The images were captured by LSM 510 Meta confocal microscopes.

### 2.8. Histological Analysis

Tissues were fixed in 10% formalin for 24 h and then processed for hematoxylin and eosin (H&E) staining. The cells were observed using the normal optical microscope. Histological analysis was performed and scored by a special pathologist according to principle of blind trial.

### 2.9. Bioinformatics Analysis of the Relationship between INF Signaling Pathway and ZIKV Infection

GSEA (gene set enrichment analysis) was used to analyze GSE97919 data set (https://http://www.ncbi.nlm/. http://nih.gov/geo) to find out the pathway or related differential genes after ZIKV infection. The interaction distance of gene network among IFN-*α* pathway, negative regulation of autophagy, and positive regulation of autophagy were calculated, and the genes with effective strong interaction among pathways were screened according to the related characteristics of topology of gene network between pathways (Betweenness Central Distribution, Harmonic Closeness Central Distribution). The interaction rate between pathways was calculated to get the IFN-*α* dominantly interacted pathway.

### 2.10. Statistical Analysis

All experiments were performed in triplicates and repeated at least three times. Data were analyzed by GraphPad Prism software 6.0 and presented as means ± SD. Group means were compared by one-way ANOVA. Differences were accepted as significant when ^∗^*p* < 0.05, ^∗∗^*p* < 0.01, and ^∗∗∗^*p* < 0.001.

## 3. Results

### 3.1. ZIKV Infection Induces Immune Cell Infiltration in SJL Mice

**Figure 1:**
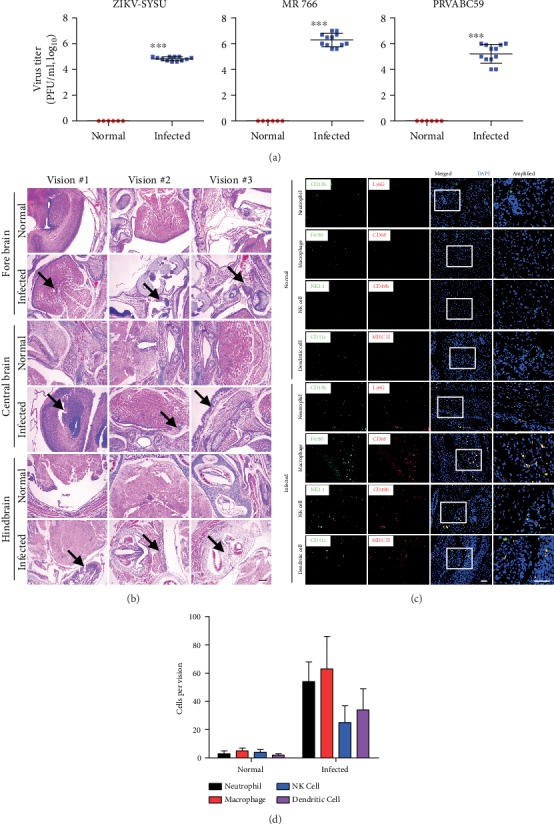
(a) ZIKV-infected pregnant mouse model with different strains at 1 × 10^6^ PFU per mice. The viral burden in neonatal mice was detected using PFU assay the first day after birth. *N* = 12, data expressed as means ± SD, ^∗∗∗^*p* < 0.005. (b) Forebrain, midbrain, and hindbrain tissues of the neonatal mice infected with ZIKV-MR766 were sectioned for H&E staining. Arrows indicates the morphological changes. Scale bar = 100 *μ*m. (c) Hindbrain tissues were sectioned as above for immunostaining with CD11b, Ly6G, and other different antibodies to detect immune cells recruitment or infiltration in neonatal mice. Scale bar = 20 *μ*m. Data are shown as representative from 12 mice. (d) Statistic results of accumulated immune cells in each vision.

To assess whether infection by ZIKV can induce severe immune response, SJL mice that were seven days pregnant were used for tail intravenous injection with different ZIKV strains. When mice were born the first day, we detected the viral burden in the brain of the suckling mouse and found that all neonatal mice were infected with different ZIKV stains (Figure 1(a)). Next, we used the ZIKV MR766 strain which is a commonly used viral strains in our following experiments. We paraffin embedded and sectioned the forebrain, midbrain, and hindbrain for histological analysis. ZIKV infection seems to induce tissue injury (atrophy, inflammation like inflammatory cell infiltration) in the mouse brain, but not severe as observed in three different fields where the morphological changes were indicated using arrows (Figure 1(b)). We chose hindbrain tissues and sectioned them for immunostaining to analyze neutrophil, macrophage, NK cell, and dendritic cells. Compared with the normal group, all these innate immune cells were shown to be accumulated in the infection group, and the accumulation of macrophage is most obvious (Figures 1(c) and 1(d)). Our results demonstrate that maternal ZIKV infection could induce immune cell accumulation in the brain of filial generation, although we are not sure whether these are resident or infiltrating cells.

### 3.2. The Progression of ZIKV Infection in Neonatal Mice

**Figure 2:**
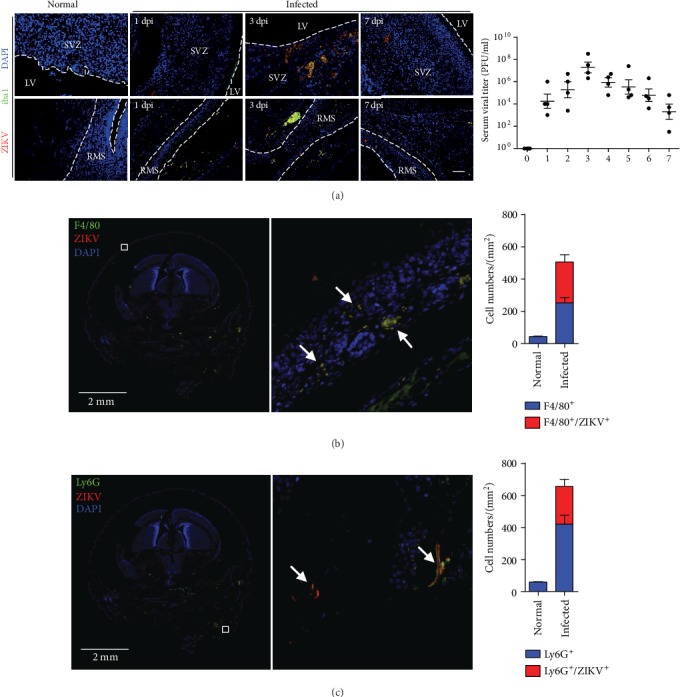
Phagocytes colocalized with ZIKV in brain. (a) The progression of ZIKV infection in neonatal mice in a time dependent manner. Iba1 and ZIKV were detected by immunofluorescence. PFU assay was performed by viral titer experiment. Scale bar = 20 *μ*m. (b) Hindbrain tissues were sectioned for immunostaining at 3 DPI with CD11b, Ly6G, and ZIKV E-protein antibodies, separately. (c) Colocalization of the cells (yellow) in the whole panoramic field was counted and compared, respectively. Scale bar = 2 mm. Data are representative from 3 independent experiment.

To explore the time point for most serious pathological changes upon ZIKV infection, we intravenously injected the virus directly to the neonatal mice as these mice are generally susceptible to viral infections due to their immature immune system. At three days postinfection (DPI), the PFU assay showed the infection level peaked and the greatest pathological change was observed, showing an increased colocalization of Iba1 and ZIKV in the subventricular zone (SVZ) and rostral migratory stream (RMS) (Figure 2(a)). The innate immune system is the first line of defense against viral or bacterial infection. The cells and molecules of innate immunity will rapidly be activated by encountering danger signals, leading to inflammation. Thus, we mainly focused on innate immune cells, especially macrophage and neutrophil. We found both macrophage and neutrophils colocalized with invaded ZIKV, which is determined by immunostaining in the brain tissue (Figures 2(b) and 2(c)). Our results indicate that phagocytes may play a crucial role in ZIKV infection *in vivo*.

### 3.3. ZIKV Infection Induces Autophagy *In Vivo*

**Figure 3:**
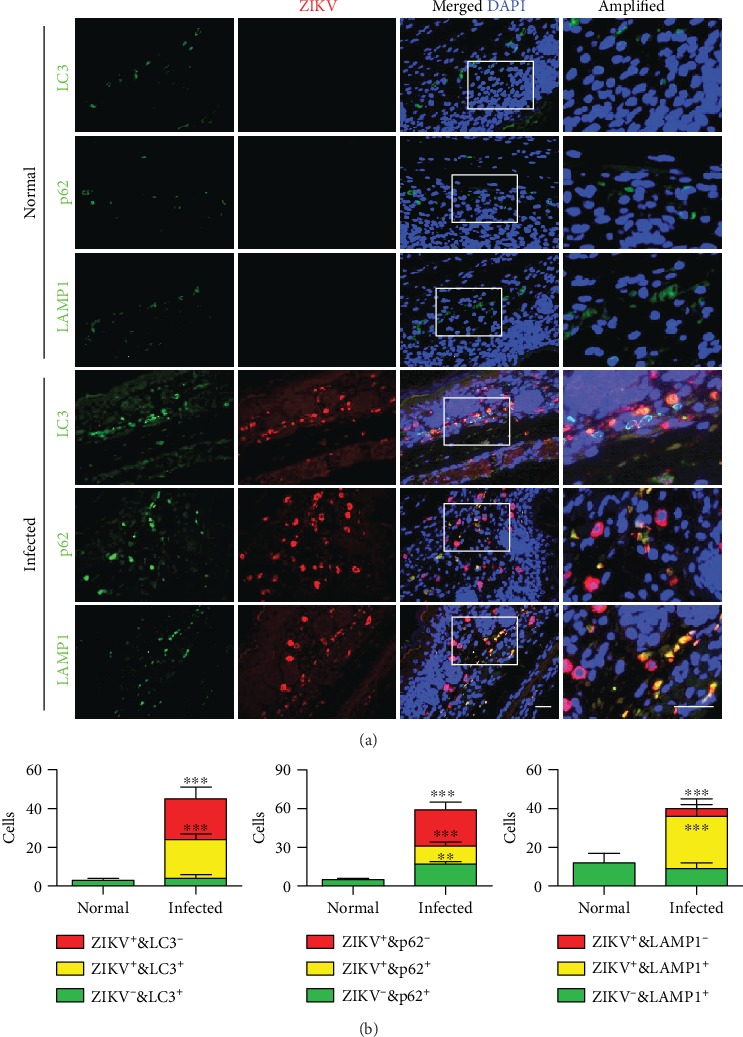
(a, b) Hindbrain tissue was immunostained with ZIKV E-protein, LC3, p62, and LAMP1 antibodies. Data are representative of three experiments and expressed as means + SD. Scale bar = 20 *μ*m. One-way ANOVA (Tukey's post hoc), ^∗^*p* < 0.05, ^∗∗^*p* < 0.01, and ^∗∗∗^*p* < 0.005.

We previously found that autophagic clearance of invaded bacteria plays a fundamental role in host immunity against pathogenic infection [15, 29]. Here, we sectioned the hindbrain for immunostaining with ZIKV E-protein, LC3, p62, and LAMP1 antibodies. Red fluorescence is the specific dye of ZIKV, and green fluorescent dyes are the specific dyes of LC3, p62, and LAMP1. Microtubule-associated protein 1A/1B-light chain 3 (LC3) is a marker protein on the autophagy membrane, p62 is a selective substrate for autophagy [28], and lysosomal-associated membrane protein 1 (LAMP-1) belongs to a family of lysosome-associated membrane glycoproteins that can be used to analyze the process of autophagy, which is the fusion of autophagosomes with lysosomes [29]. The colocalization of autophagic proteins (which have more LC3, p62, and LAMP1 puncta) with ZIKV E-proteins suggests the occurrence of autophagy in ZIKV-infected cells (Figure 3(a)). Compared with the normal group, ZIKV his distributed in the whole hindbrain tissue, and the high rate of virus-positive cells indicates that ZIKV has a strong infectious ability (Figures 3(a) and 3(b)). Inducible properties of autophagy are mainly manifested in two aspects. The rapid synthesis of autophagy-related proteins, and the rapid and massive formation of autophagosomes was found. After finding that ZIKV infection could induce LC3dots *in vivo*, we further tried to determine the changes in autophagosome formation upon ZIKV infection.

### 3.4. ZIKV Infection Resulted in Elevated Autophagosome Formation *In Vitro*

**Figure 4:**
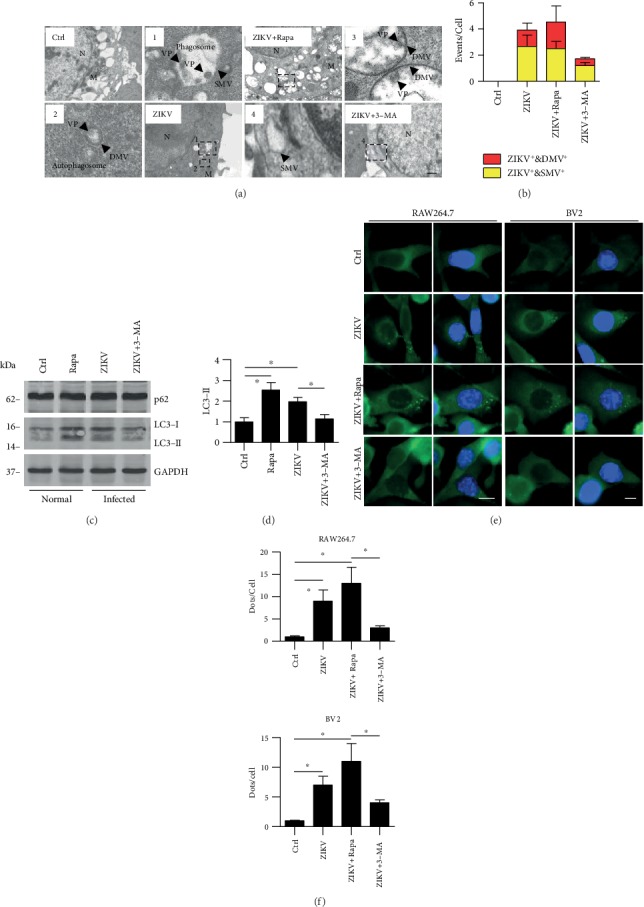
ZIKV infection led to increased autophagosome formation *in vitro*. (a) RAW264.7 cells were infected with ZIKV for 1 hour (MOI = 10 : 1). Before infection, the cells were also treated with rapamycin and 3-MA; rapamycin was used at 3 *μ*M (12 h), and 3-MA was used at 3 mM (3 h). After infection, cells were processed and examined by TEM. The areas of 1-4 were expanded, SMV represents phagosome, DMV indicates autophagosome, and we speculate that VP (form the morphology and size) may represent virus particle. Scale bar = 2 *μ*m. (b) The number of autophagosome and phagosome in each cell was counted, 100 cells in each sample, respectively. Data are representative of three experiments with similar results. (c, d) Western blotting was performed to detect the transition of LC3 upon ZIKV infection in RAW264.7 cells. One-way ANOVA; Tukey's posthoc test, ^∗^*p* < 0.05. (e) RAW264.7 and BV2 were infected with ZIKV for 1 hour (MOI = 10 : 1). Before infection, the cells were also treated with rapamycin or 3-MA as above. (f) The visible LC3-GFP puncta in each cell were counted. Values are from 100 cells/sample. *Means* ± *SD*. One-way ANOVA; Tukey's post hoc test, ^∗^*p* < 0.05.

Autophagosome has two characteristics: one is a bilayer membrane and the other is a cytoplasmic component, such as mitochondria and endoplasmic reticulum debris. Phagosome is a single-membrane vesicle (SMV), and autophagosome is a double-membrane vesicle (DMV). Transmission electron microscopy (TEM) shows the accumulation of visible autophagosomes with double membranes (Figure 4(a)). Compared with untreated RAW264.7 cells, autophagosomes were found to be increased in ZIKV-infected cells; likewise, rapamycin which is the positive control also significantly increased the formation of autophagosome. Besides, ZIKV inducing the formation of autophagosome was inhibited by 3-MA which is the negative control (Figures 4(a) and 4(b)). Therefore, from the morphological evidence, we can further demonstrate that ZIKV infection induced the process of autophagy and this result is consistent with the above results which showed the recruitment of LC3 punctuation (Figures 4(a) and 4(b)). Our finding is consistent with the previous studies that confirmed the colocalization of the virus and viral replication complexes (invaginated vesicles) [31, 32], although we do not know whether ZIKV could replicate in autophagosomes.

In addition, we determined the LC3 transmission using western blotting and found that LC3-II has a significant increase in autophagic cells or upon ZIKV infection which is consistent with the above results (Figures 4(c) and 4(d)). While LC3 transformation happens, there is no obvious difference of p62 upon ZIKV infection, which means the degradation process and the ubiquitination will be still ongoing afterward (Figure 4(c)). Moreover, the transformation of LC3-I to LC3-II was inhibited in 3-MA-treated cells upon ZIKV infection (Figures 4(c) and 4(d)). A previous report has shown that ZIKV is associated with severe neural development impairments [33]. To further determine whether ZIKV infection can induce autophagy *in vitro*, we used another type of neuroglia cells, BV2, to perform LC3 punctuation using immunostaining. Using rapamycin as an autophagy activator or positive control and 3-MA as an autophagy inhibitor or negative control, respectively, we found that compared with the control group, ZIKV infection increased LC3 punctuation as shown by confocal microscopy images (Figures 4(e) and 4(f)). Both morphological and molecular data indicate that ZIKV induces autophagy *in vitro*.

### 3.5. Autophagy Regulates ZIKV Clearance in Macrophages

**Figure 5:**
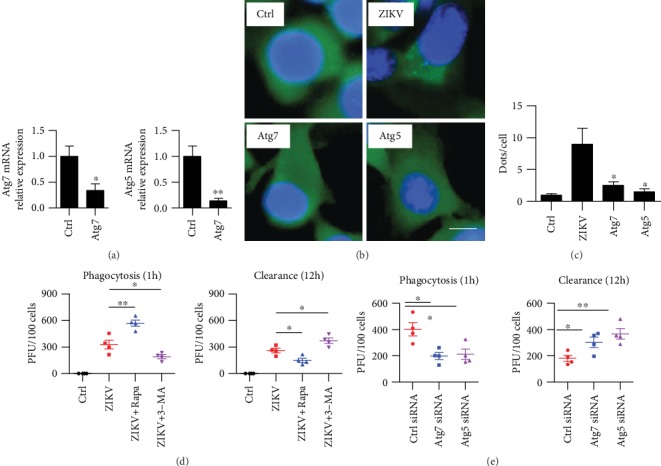
Autophagy promoted ZIKV clearance in RAW264.7 cells. (a) Raw264.7 cells were transfected with ctrl siRNA or Atg7 and Atg5 siRNAs. qRT-PCR was performed for knockdown efficiency test. Means + SD from triplicate. ^∗^*p* < 0.05, ^∗∗^*p* < 0.01. (b) Cells were infected with ZIKV for 1 hour, (MOI = 10 : 1). Before infection, the cells were also transfected with LC3-GFP and treated with Atg5 siRNA and Atg7 siRNA. Immunostaining was performed to detect LC3 puncta. (c) Puncta number in each cell was counted. Values are means ± SD from 20 cells/sample. One-way ANOVA; Tukey's post hoc test, ^∗^*p* < 0.05. (d) RAW264.7 cells were infected with ZIKV for 1 hour (MOI = 10 : 1). Before infection, the cells were also treated with rapamycin and 3-MA; the rapamycin was used at (3 *μ*M, 12 hours); 3-MA was used at (3 mM, 3 hours). The number of internalized virus per cell after 1 h infection was counted by PFU assay. After infection for 12 h, the unbound virus was washed away and fresh medium was added. The clearance assay was performed as counting the number of internalizing virus per cell by PFU assay. (e) Before infection, RAW264.7 cells were treated with negative control siRNA, Atg5 siRNA, and Atg7 siRNA. The number of internalized virus per cell was counted as above. Data were representative of three experiment results. Means ± SD. One-way ANOVA; Tukey's post hoc test, ^∗^*p* < 0.05, ^∗∗^*p* < 0.01.

Some studies have demonstrated that autophagy is an important defense mechanism to clear intracellular pathogenic bacteria [34]. It is well known that Atg5 and Atg7 are critical for autophagy [30]. So, the occurrence of autophagy is difficult in Atg5- or Atg7-deficient cells. We found that autophagy was weakened in Atg5 siRNA- and Atg7 siRNA-treated cells upon ZIKV infection (Figures 5(a)–5(c)). Next, we tried to find out whether autophagy plays a role in phagocytosis or clearance in macrophage infected with ZIKV. RAW264.7 cells were pretreated with rapamycin or 3-MA, then infected with ZIKV for 1 hour. We detected phagocytosis by counting the invaded virus number using PFU assay. The number of ZIKV was increased in the rapamycin treatment group, while the intracellular virus count was decreased in the 3-MA-treated group (Figure 5(d), left panel). We detected the number of viruses after 12 hours and found that the intracellular virus count was increased in the 3-MA-treated group because the autophagy was blocked which means the clearance ability was inhibited (Figure 5(d), right panel). We then manipulated autophagy (Atg5 siRNA or Atg7 siRNA) and found that the phagocytized viruses were decreased in autophagy-deficient cells (Figure 5(e)). Besides, the clearance ability of macrophage against ZIKV infection was also inhibited (Figure 5(e)). These data indicated that the induction of autophagy strengthened host immunity to resist this pathogen. Taken together, our data identified that ZIKV-induced autophagy played a crucial role in phagocytes by accelerating viral phagocytosis and clearance.

### 3.6. Type I IFN Participates in Autophagic Immunity upon ZIKV Infection

**Figure 6:**
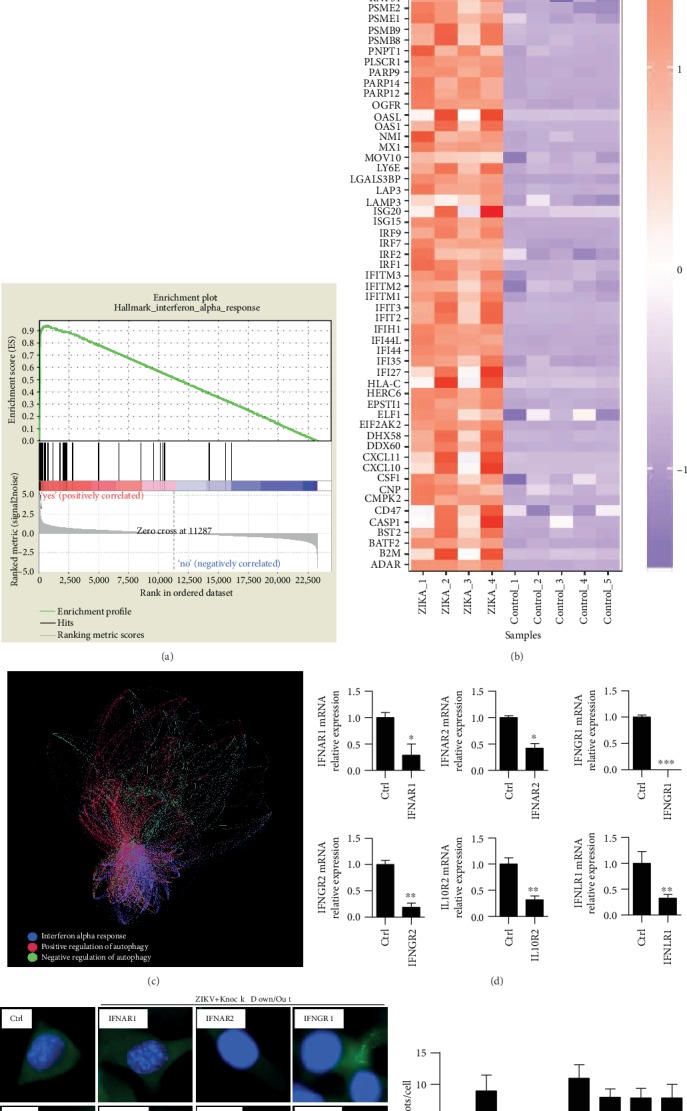
(a) GSE97919 Zika data set. GSEA (gene set enrichment analysis) shows there was a positive correlation between ZIKV infection and IFN-*α* pathway activation. Enrichment score = 0.9344. (b) Different genes enriched by IFN-*α* activation upon ZIKV infection, and 70 IFN-*α* pathway genes were upregulated. (c) Analysis of interaction among IFN-*α* pathway, negative regulation of autophagy, and positive regulation of autophagy. (d) RAW264.7 cells were transfected with IFNAR1 siRNA, IFNAR2 siRNA, IFNGR1 CRISPR-Cas9, IFNGR2 siRNA, IFNLR1 siRNA, or IL10R2 siRNA, respectively. qRT-PCR was performed to test the knockdown or knockout efficiency. (e) Cells above were cultured and transfected with LC3-GFP, then infected with ZIKV, MOI = 10 : 1. LC3 fluoresce was observed using confocal microscopy. (f) LC3 puncta numbers in 100 cells of each sample were counted. Means ± SD. Data were representative of three experiment results.

Type I interferon (IFN) signaling plays a particularly important role against viral infection. It is mainly through cell surface pattern recognition receptors that make the cells produce antiviral proteins. Through GSEA analysis of the ZIKV chip, we found that after ZIKV infection, IFN-*α* signaling pathway was significantly enriched (nominal *p* value = 0, FDR = 0.0159) (Supplementary Table 1), and 70 IFN-*α* pathway genes were significantly upregulated (Figures 6(a) and 6(b)). In addition, ZIKV infection and IFN-*α* pathway activation have a significant positive correlation trend with an enrichment score = 0.9344 (Figure 6(a)). However, upon ZIKV infection, intracellular receptors play more important roles in mediating the balance of autophagic immunity. Through the analysis of the disease pathway interaction, we found that after ZIKV infection, there were 1825 pairs of effective interaction genes between the IFN-*α* pathway and positive regulation of autophagy, with an effective interaction rate = 43% (Figure 6(c)). At the same time, the effective interaction rate between the IFN-*α* pathway and negative regulation of autophagy was 30% (Supplementary Table 2). Therefore, there was a dominant interaction between the IFN-*α* pathway and positive regulation of autophagy. After IFN-*α* pathway is activated, it can effectively activate and promote autophagy [35]. We believe that after ZIKV infection, IFN-*α* signaling pathway is strongly activated and has a positive correlation trend with autophagy. Next, we further explored and found that ZIKV-induced autophagy was blocked in the RAW264.7 cells treated with IFNAR1 siRNA and IFNAR2 siRNA as shown with increased ration of LC3 punctuation, but not in cells treated with IFNGR1 CRISPR-Cas9, IFNGR2 siRNA, IFNLR1 siRNA, and IL10R2 siRNA groups which showed no significant difference (Figures 6(d)–6(f)). Taken together, our results identified that ZIKV infection-induced type I IFN signaling correlated with autophagy.

### 3.7. Phagocytes Contribute to Autophagic Clearance against ZIKV Infection *In Vivo*

**Figure 7:**
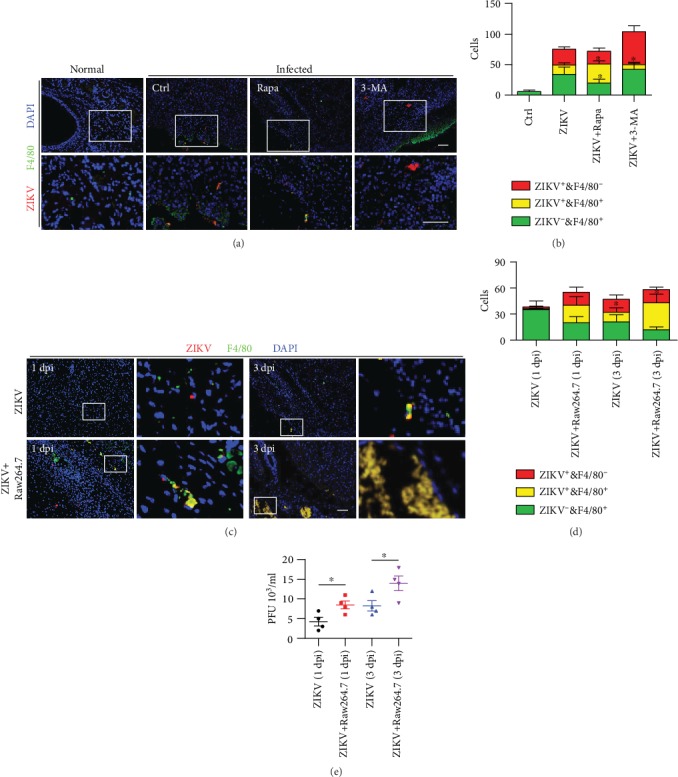
Phagocytes contribute to autophagic clearance against ZIKV infection *in vivo*. (a) Neonatal mice were treated with rapamycin (3 mg/kg) and 3-MA (3 mg/kg) and then subcutaneously injected with ZIKV. Then, the mice were sacrificed and brain tissues were sectioned for immunostaining with ZIKV E-protein and F4/80 antibody. (b) ZIKV and F4/80 positive cells were counted in each vision. (c) Neonatal mice were infected with ZIKV or ZIKV-infected RAW264.7 cells, respectively. Three days after infection, the mice were sacrificed and brain tissues were sectioned for immunostaining with ZIKV E-protein and F4/80 antibody. Scale bar = 20 *μ*m. (d) ZIKV- and F4/80-positive cells were counted. (e) The number of virus in the brain was counted using PFU assay. Means ± SD. One-way ANOVA; Tukey's post hoc test, ^∗^*p* < 0.05. Data were representative from three mice of each experiment.

Having confirmed that macrophage played an important role against ZIKV, we further determined whether the manipulation of autophagy affect the functions of macrophage *in vivo*. We found that rapamycin-treated mice have lower distribution of ZIKV as compared with the control group, while 3-MA has a negative role in mediating ZIKV infection (Figures 7(a) and 7(b)). Mice treated with either rapamycin or 3-MA has no discernible behavior differences as compared with normal mice. Similarly, the accumulation of ZIKV- and F4/80-positive cells was significantly increased in rapamycin-treated mice upon ZIKV infection (Figures 7(a) and 7(b)). Next, we tried to find out whether macrophage could help the translocation of the ZIKV. Using ZIKV and ZIKV-infected RAW264.7 cells to infect mice in a time-dependent manner, we found the most severe pathological change 3 days postinfection, the time point when infection was observed most serious as before (Figures 7(c)–7(e)). These data proved again that ZIKV infection exerted an important influence on macrophage; the adoption of macrophage *in vivo* accelerates the transmission of ZIKV that is beneficial to host defenses. We believe that the movability of the macrophage could help the spread of ZIKV. Like autophagy, macrophage plays a balanced role in the immune response against ZIKV infection.

## 4. Discussion

Autophagy is a complex and tightly regulated cellular pathway responsible for the lysosomal degradation of long-lived proteins, cellular organelles, and parts of the cytosol and has been involved in important defense mechanism against virus infection. It has been reported that autophagy plays important roles during flavivirus infection [36–39]. However, the biology and the pathogenesis of ZIKV still need further exploration.

Scientists have demonstrated that ZIKV is capable of infecting human skin fibroblast cells and human pluripotent stem cell- (hPSC-) derived neural progenitor cells (NPCs) *in vitro*, which induced apoptotic cell death [39, 40]. ZIKV can cross the placenta membrane causing microcephaly through targeting cortical progenitor cells by apoptosis and autophagy [41]. After ZIKV infection in human trophoblasts (CTBs), LC3 converts from the soluble form LC3-I to the lipidated form LC3-II significantly increasing at 6 hours and 12 hours postinfection [42]. ZIKV-infected placental at 5 days postinfection showed a high expression of LC3 with a decrease in p62, a substrate degraded by autophagy pathway is negatively related to autophagy [42] which demonstrated that ZIKV infection induces canonical autophagy response.

Flaviviruses, including ZIKV, induce invaginations of the ER giving rise to clusters of vesicle-designated vesicle packets or double-membrane vesicles (DMVs), which are associated with viral genome replication [43–46]. Akt phosphorylation at Thr308 and Ser473 is required for its full kinase activity, and Akt-mediated mTOR phosphorylation at Ser2448 is essential for autophagy [47]. ZIKV nonstructural (NS) 4A and NS4B inhibit the Akt/mTOR signaling pathway by decreasing Akt phosphorylation at both Thr308 and Ser473 and activate autophagy [48]. Further study shows that to maintain normal homeostasis, cells with ZIKV infection regulate the ER by reticulophagy, a selective form of autophagy that leads to fragmentation of the ER and subsequent lysosomal degradation but the precise signaling pathways that account for the induction of selective autophagy are still unknown currently [49]. Besides, mosquito saliva could regulate the host inflammatory immune responses [50], and the mosquito salivary protein promotes Zika virus transmission by activation of autophagy in host immune cells of the monocyte lineage [51]. In addition, BPI Fold Containing Family B Member 3 (BPIFB3) as a regulator of autophagy positively regulates ZIKV infection and promotes the formation of viral replication [52]. Because viruses are intracellular pathogens, autophagy is a defined antiviral response that is partly regulated by nutritional and STING-mediated signaling [53]. And Drosophila has revealed that insects are able to utilize autophagy to respond to ZIKV in neuronal tissues [26].

ZIKV induces an innate antiviral response. Type I, Type II, and Type III IFN protection against ZIKV infection. Toll-like receptor 3 (TLR3) exerts antiviral effects against ZIKV-induced innate antiviral responses in primary human skin fibroblasts [40]; inhibition of TLR3 expression results in a strong increase in the viral RNA copy number 48 h, reminiscent to the activation of TLR3 related to the pathway of autophagy. Also, we found antiviral infection effects associated with type-I interferon; however, transgenic mice were not used due to limited condition for breeding IFN-*α*R2 double knockout homozygote. The classical intracellular signaling pathway of autophagy involving relating genes, Atg5/Atg7, is confirmed in our experiment. Type I IFN signaling is activated to make the host more resistant to ZIKV invasion. But whether autophagy is an important part of viral clearance is rarely reported. Our study offers new insights into the mechanism that ZIKV-induced autophagy can accelerate viral transmission and clearance *in vivo* and *in vitro*. Interestingly, the viral burden of the placenta in Atg16L1-deficient mice infected with ZIKV was 10-fold lower compared with WT controls. ZIKV infection induces autophagy *in vivo*, whereas loss of Atg16l1 expression impairs the intrauterine transmission of ZIKV [42]. In a previous study, three compounds including quinacrine (QC), mefloquine (MQ), and GSK369796 had high anti-DENV activity in the DENV2 replicon in the analysis of their antiviral activities by qRT-PCR and showed antiviral activity against the rapidly emerging ZIKV [54].

Taken together, autophagy in macrophage is an important part of ZIKV's early infection. On the one hand, macrophages can engulf the virus; on the other hand, the activity of macrophages also helps the spread of the virus. Like autophagy, macrophage plays a balanced role in the immune response against ZIKV infection. And autophagy is like a wider range of phagocytosis. Viruses and phagocytic cells are constantly playing games. Once the balance is broken, the ultimate result will be the extreme phenomena. Either the virus is cleared or virus is completely invaded, resulting in a more serious illness. These phenomena are not uncommon in the process of innate immunity. Nevertheless, a series of innate immune defenses triggered by the interaction between autophagy and ZIKV infection has not been thoroughly studied. As ZIKV has seriously damaged global public health and because of the lack of effective treatment, further study is required for the mechanism about how ZIKV is involved in autophagy which reveals the role of autophagy in immunity to help develop and design effective therapeutic drugs to control viral infections and treat diseases.

## Data Availability

The data used to support the findings of this study are available from the corresponding author upon request.

## Supplementary Materials

Supplementary Materials Supplementary Table 1: through gene set enrichment analysis of the Zika Virus chip, it was found that after ZIKV infection, the IFN-*α* signaling pathway was significantly enriched (normalized enrichment score (NES) = 1.936412). And the *p* value was corrected with multiple testing (nominal *p* value = 0, false discovery rate (FDR) *q* value = 0.0159, and per-family error rate (PFER) *p* value = 0.047). Supplementary Table 2: gene association analysis was performed on the gene sets of three pathways in the Zika Virus chip. The result found that there were 1272 genes that effectively interacted between the IFN-*α* pathway and the negative autophagy pathway. The effective interaction rate between IFN-*α* pathway and negative regulation of autophagy was 30%.

## References

[1] Dick G. W. A., Kitchen S. F., Haddow A. J. (1952). Zika virus. I. Isolations and serological specificity. *Transactions of the Royal Society of Tropical Medicine and Hygiene*.

[2] Simpson D. I. (1964). Zika virus infection in man. *Transactions of the Royal Society of Tropical Medicine and Hygiene*.

[3] Sariol C. A., Nogueira M. L., Vasilakis N. (2018). A tale of two viruses: does heterologous Flavivirus immunity enhance Zika disease?. *Trends in Microbiology*.

[4] Duffy M. R., Chen T. H., Hancock W. T. (2009). Zika virus outbreak on Yap Island, Federated States of Micronesia. *The New England Journal of Medicine*.

[5] Rozé B., Najioullah F., Signate A. (2016). Zika virus detection in cerebrospinal fluid from two patients with encephalopathy, Martinique, February 2016. *Eurosurveillance*.

[6] Brasil P., Pereira J. P., Moreira M. E. (2016). Zika virus infection in pregnant women in Rio de Janeiro. *The New England Journal of Medicine*.

[7] Sharp T. M., Muñoz-Jordán J., Perez-Padilla J. (2016). Zika virus infection associated with severe thrombocytopenia. *Clinical Infectious Diseases*.

[8] Kodati S., Palmore T. N., Spellman F. A., Cunningham D., Weistrop B., Sen H. N. (2017). Bilateral posterior uveitis associated with Zika virus infection. *Lancet*.

[9] Mécharles S., Herrmann C., Poullain P. (2016). Acute myelitis due to Zika virus infection. *Lancet*.

[10] Carteaux G., Maquart M., Bedet A. (2016). Zika virus associated with meningoencephalitis. *The New England Journal of Medicine*.

[11] Calvet G., Aguiar R. S., Melo A. S. O. (2016). Detection and sequencing of Zika virus from amniotic fluid of fetuses with microcephaly in Brazil: a case study. *The Lancet Infectious Diseases*.

[12] Mlakar J., Korva M., Tul N. (2016). Zika virus associated with microcephaly. *The New England Journal of Medicine*.

[13] Kleber de Oliveira W., Cortez-Escalante J., de Oliveira W. T. G. H. (2016). Increase in reported prevalence of microcephaly in infants born to women living in areas with confirmed Zika virus transmission during the first trimester of pregnancy-Brazil, 2015. *MMWR. Morbidity and Mortality Weekly Report*.

[14] Deretic V., Saitoh T., Akira S. (2013). Autophagy in infection, inflammation and immunity. *Nature Reviews. Immunology*.

[15] Li X., He S., Zhou X. (2016). Lyn delivers bacteria to lysosomes for eradication through TLR2-initiated autophagy related phagocytosis. *PLoS Pathogens*.

[16] Levine B., Mizushima N., Virgin H. W. (2011). Autophagy in immunity and inflammation. *Nature*.

[17] Shoji-Kawata S., Levine B. (2009). Autophagy, antiviral immunity, and viral countermeasures. *Biochimica et Biophysica Acta*.

[18] Munz C. (2009). Enhancing immunity through autophagy. *Annual Review of Immunology*.

[19] Choi Y., Bowman J. W., Jung J. U. (2018). Autophagy during viral infection - a double-edged sword. *Nature Reviews. Microbiology*.

[20] Liang Q., Chang B., Brulois K. F. (2013). Kaposi's sarcoma-associated herpesvirus K7 modulates Rubicon-mediated inhibition of autophagosome maturation. *Journal of Virology*.

[21] Liang Q., Chang B., Lee P. (2015). Identification of the essential role of viral Bcl-2 for Kaposi's sarcoma-associated herpesvirus lytic replication. *Journal of Virology*.

[22] Williams L. R., Taylor G. S. (2012). Autophagy and immunity - insights from human herpesviruses. *Frontiers in Immunology*.

[23] Peng H., Liu B., Yves T. (2018). Zika virus induces autophagy in human umbilical vein endothelial cells. *Viruses*.

[24] Kriegstein A., Alvarez-Buylla A. (2009). The glial nature of embryonic and adult neural stem cells. *Annual Review of Neuroscience*.

[25] Souza B. S. F., Sampaio G. L. A., Pereira C. S. (2016). Zika virus infection induces mitosis abnormalities and apoptotic cell death of human neural progenitor cells. *Scientific Reports*.

[26] Liu Y., Gordesky-Gold B., Leney-Greene M., Weinbren N. L., Tudor M., Cherry S. (2018). Inflammation-induced, STING-dependent autophagy restricts Zika virus infection in the drosophila brain. *Cell Host Microbe*.

[27] Gratton R., Agrelli A., Tricarico P. M., Brandao L., Crovella S. (2019). Autophagy in Zika virus infection: a possible therapeutic target to counteract viral replication. *International Journal of Molecular Sciences*.

[28] Pan T., Peng Z., Tan L. (2018). Nonsteroidal anti-inflammatory drugs potently inhibit the replication of Zika viruses by inducing the degradation of AXL. *Journal of Virology*.

[29] Li X., Ye Y., Zhou X., Huang C., Wu M. (2015). Atg 7 enhances host defense against infection via downregulation of superoxide but upregulation of nitric oxide. *Journal of Immunology*.

[30] He S., Li X., Li R. (2016). Annexin A2 modulates ROS and Impacts inflammatory response via IL-17 signaling in polymicrobial sepsis mice. *PLoS Pathogens*.

[31] Taguwa S., Yeh M. T., Rainbolt T. K. (2019). Zika Virus Dependence on Host Hsp70 Provides a Protective Strategy against Infection and Disease. *Cell Reports*.

[32] Zheng Y., Liu Q., Wu Y. (2018). Zika virus elicits inflammation to evade antiviral response by cleaving cGAS via NS1-caspase-1 axis. *The EMBO Journal*.

[33] Garcez P. P., Loiola E. C., Madeiro da Costa R. (2016). Zika virus impairs growth in human neurospheres and brain organoids. *Science*.

[34] Yang X., Ye H., He M. (2018). LncRNA PDIA3P interacts with c-Myc to regulate cell proliferation via induction of pentose phosphate pathway in multiple myeloma. *Biochemical and Biophysical Research Communications*.

[35] Schmeisser H., Bekisz J., Zoon K. C. (2014). New function of type I IFN: induction of autophagy. *Journal of Interferon & Cytokine Research*.

[36] Heaton N. S., Randall G. (2010). Dengue virus-induced autophagy regulates lipid metabolism. *Cell Host & Microbe*.

[37] McLean J. E., Wudzinska A., Datan E., Quaglino D., Zakeri Z. (2011). Flavivirus NS4A-induced autophagy protects cells against death and enhances virus replication. *The Journal of Biological Chemistry*.

[38] Metz P., Chiramel A., Chatel-Chaix L. (2015). Dengue virus inhibition of autophagic flux and dependency of viral replication on proteasomal degradation of the autophagy receptor p 62. *Journal of Virology*.

[39] Tang H., Hammack C., Ogden S. C. (2016). Zika virus infects human cortical neural progenitors and attenuates their growth. *Cell Stem Cell*.

[40] Hamel R., Dejarnac O., Wichit S. (2015). Biology of Zika virus infection in human skin cells. *Journal of Virology*.

[41] Cugola F. R., Fernandes I. R., Russo F. B. (2016). The Brazilian Zika virus strain causes birth defects in experimental models. *Nature*.

[42] Cao B., Parnell L. A., Diamond M. S., Mysorekar I. U. (2017). Inhibition of autophagy limits vertical transmission of Zika virus in pregnant mice. *Journal of Experimental Medicine*.

[43] Junjhon J., Pennington J. G., Edwards T. J., Perera R., Lanman J., Kuhn R. J. (2014). Ultrastructural characterization and three-dimensional architecture of replication sites in dengue virus-infected mosquito cells. *Journal of Virology*.

[44] Miorin L., Romero-Brey I., Maiuri P. (2013). Three-dimensional architecture of tick-borne encephalitis virus replication sites and trafficking of the replicated RNA. *Journal of Virology*.

[45] Gillespie L. K., Hoenen A., Morgan G., Mackenzie J. M. (2010). The endoplasmic reticulum provides the membrane platform for biogenesis of the flavivirus replication complex. *Journal of Virology*.

[46] Welsch S., Miller S., Romero-Brey I. (2009). Composition and three-dimensional architecture of the dengue virus replication and assembly sites. *Cell Host & Microbe*.

[47] Chan C. H., Jo U., Kohrman A. (2014). Posttranslational regulation of Akt in human cancer. *Cell & Bioscience*.

[48] Liang Q., Luo Z., Zeng J. (2016). Zika virus NS4A and NS4B proteins deregulate Akt-mTOR signaling in human fetal neural stem cells to inhibit neurogenesis and induce autophagy. *Cell Stem Cell*.

[49] Lennemann N. J., Coyne C. B. (2017). Dengue and Zika viruses subvert reticulophagy by NS2B3-mediated cleavage of FAM134B. *Autophagy*.

[50] Jin L., Guo X., Shen C. (2018). Salivary factor LTRIN from Aedes aegypti facilitates the transmission of Zika virus by interfering with the lymphotoxin-*β* receptor. *Nature Immunology*.

[51] Sun P., Nie K., Zhu Y. (2020). A mosquito salivary protein promotes flavivirus transmission by activation of autophagy. *Nature Communications*.

[52] Evans A. S., Lennemann N. J., Coyne C. B. (2020). BPIFB3 regulates ER morphology to facilitate flavivirus replication. *Journal of Virology*.

[53] Moretti J., Roy S., Bozec D. (2017). STING senses microbial viability to orchestrate stress-mediated autophagy of the endoplasmic reticulum. *Cell*.

[54] Balasubramanian A., Teramoto T., Kulkarni A. A., Bhattacharjee A. K., Padmanabhan R. (2017). Antiviral activities of selected antimalarials against dengue virus type 2 and Zika virus. *Antiviral Research*.

