# Supplementary material for: Autophagy Contributes to Host Immunity and Protection against Zika Virus Infection via Type I IFN Signaling
Source: Mediators Inflamm. 2020 Apr 28;2020:9527147. doi: 10.1155/2020/9527147 (PMC7204160; doi:10.1155/2020/9527147)
Supplement: Supplementary Materials — Supplementary Table 1: through gene set enrichment analysis of the Zika Virus chip, it was found that after ZIKV infection, the IFN-α signaling pathway was significantly enriched (normalized enrichment score (NES) = 1.936412). And the p value was corrected with multiple testing (nominal p value = 0, false discovery rate (FDR) q value = 0.0159, and per-family error rate (PFER) p value = 0.047). Supplementary Table 2: gene association analysis was performed on the gene sets of three pathways in the Zika Virus chip. The result found that there were 1272 genes that effectively interacted between the IFN-α pathway and the negative autophagy pathway. The effective interaction rate between IFN-α pathway and negative regulation of autophagy was 30%. [file 9527147.f1.docx]

| Gene Set | Interferon α Response |
| --- | --- |
| Enrichment Score (ES) | 0.934431 |
| Normalized Enrichment Score (NES) | 1.936412 |
| Nominal p-value | 0 |
| FDR q-value | 0.01589 |
| FWER p-Value | 0.047 |

**Table 1. GSEA Result**

Through Gene Set Enrichment Analysis of Zika Virus chip, it was found that after ZIKV infection, IFN-α signaling pathway was significantly enriched (Normalized Enrichment Score (NES)= 1.936412). And p value was corrected with multiple testing (Nominal p-value=0, False discovery rate (FDR) q-value = 0.0159, Per-family error rate (PFER) p-Value = 0.047).

**Table 2. Pathway Interaction Rate**

| Pathway A | Pathway B | Effective Interaction Gene | Interaction Rate |
| --- | --- | --- | --- |
| Negative-Autophagy | Positive-Autophagy | 1121 | 0.265765766 |
| Negative-Autophagy | IFN-α | 1272 | 0.301564723 |
| Positive-Autophagy | IFN-α | 1825 | 0.432669512 |

Gene association analysis was performed on the gene sets of three pathways in Zika Virus chip. The result found that there were 1272 genes that effectively interacted between the IFN-α pathway and the Negative-Autophagy pathway. The effective interaction rate between IFN-α pathway and negative regulation of autophagy was 30%.
